# Sociodemographic, Clinical, and Psychosocial Characteristics of People with Hemophilia in Portugal: Findings from the First National Survey

**DOI:** 10.1055/s-0038-1624568

**Published:** 2018-02-14

**Authors:** Patrícia Ribeiro Pinto, Ana Cristina Paredes, Susana Pedras, Patrício Costa, Miguel Crato, Susana Fernandes, Manuela Lopes, Manuela Carvalho, Armando Almeida

**Affiliations:** 1 Life and Health Sciences Research Institute (ICVS), School of Medicine, University of Minho, Braga, Portugal; 2 ICVS/3B's – PT Government Associate Laboratory, Braga/Guimarães, Portugal; 3 School of Psychology, University of Minho, Braga, Portugal; 4 Faculty of Psychology and Education Sciences, University of Porto, Porto, Portugal; 5 Portuguese Haemophilia Association, Lisbon, Portugal; 6 European Haemophilia Consortium, Brussels, Belgium; 7 Department of Transfusion Medicine and Blood Bank, Centre of Hemophilia, Centro Hospitalar São João, Porto, Portugal

**Keywords:** hemophilia, pain, quality of life, functionality, psychological factors

## Abstract

Hemophilia is a rare genetic bleeding disorder associated with pain, impaired functionality, and decreased quality of life (QoL). Several studies have focused on patient-reported outcomes of people with hemophilia (PWH) worldwide, but no such data are available for Portugal. This survey aimed to describe sociodemographic, clinical, and psychosocial characteristics of PWH of all ages in Portugal. Questionnaires were answered by self-report or by parents of children with hemophilia (proxy version). Variables assessed were sociodemographic and clinical, physical activity patterns, pain, functionality (HAL/PedHAL), QoL (A36 Hemofilia-QoL/CHO-KLAT), anxiety and depression (PROMIS), and illness perceptions (IPQ-R). One-hundred and forty-six PWH answered the survey: 106 adults, 21 children/teenagers between 10 and 17 years, 11 children between 6 and 9 years, and 8 children between 1 and 5 years. Most participants had severe hemophilia (60.3%) and type A was most commonly reported (86.3%). Bleeding episodes, joint deterioration, and pain were very prevalent, with the ankles and knees being the most affected joints, as illustrated by HAL/PedHAL scores. The A36 Hemofilia-QoL assessment showed moderate QoL (96.45; 0–144 scale) and significant anxiety and depression symptoms were found in 36.7 and 27.2% of adults, respectively. CHO-KLAT global score (0–100 scale) was 75.63/76.32 (self-report/proxy). Concerning hemophilia-related illness beliefs, a perception of chronicity and symptoms unpredictability was particularly prominent among adults and children/teenagers. This survey provided a comprehensive characterization of Portuguese PWH, including the first report of psychosocial characteristics. The findings allow for a deeper understanding of life with hemophilia in Portugal and the identification of relevant health care and research needs.

## Introduction

Hemophilia is a rare and lifelong genetic bleeding disorder linked to the X-chromosome, which is caused by a deficiency of clotting factor, more specifically factor VIII (hemophilia A) or IX (hemophilia B). 1 The severity of hemophilia depends on clotting factor level and is classified accordingly as mild (clotting factor between 5 and 40% of normal), moderate (1–5% of normal clotting factor), or severe (clotting factor level <1% of normal). 2 The main clinical manifestation is an increased bleeding tendency, either spontaneous or related to trauma or surgery. Spontaneous bleeding episodes occur most often in the joints (hemarthrosis) and, if recurrent, might lead to hemophilic arthropathy, characterized by joint deformity, disability and chronic pain, and responsible for a variable degree of impairment and limitations in daily living, that can ultimately lead to the need for orthopaedic surgery. 3

Clotting factor replacement therapy, aiming to increase deficient factor levels, is the mainstay in hemophilia treatment, either delivered on demand (to treat a bleed after its occurrence) or prophylactically (regularly, to prevent bleeds). However, while regular prophylaxis since childhood is the recommended standard of care for bleeding prevention and related complications, its widespread adoption is hindered by a very high cost. 1 4 5 6 As early prophylaxis is relatively recent in developed countries, and still not widely accessible in developing countries, most adult PWH already present damaged joints due to the lack of prophylactic treatment in their infancy. 7 Yet, spontaneous bleeding can still happen in children under prophylaxis and trauma-related bleeds cannot be at all prevented. 8 In addition, development of alloantibodies against replacement factor VIII or IX (inhibitors) rises another serious problem, by limiting treatment options and consequently leaving patients at greater risk for hemarthrosis and hemophilic arthropathy. 1

Several studies have shown that hemophilia has a negative impact on quality of life (QoL), which is often lower among PWH, when compared with the general population. 9 10 Actually, current guidelines for optimal hemophilia care recognize the relevance of QoL and establish psychosocial health promotion as a priority in the care of PWH, along with the prevention of bleeds and management of complications. 5 Therefore, QoL should be routinely monitored in clinical practice, as part of patient care and as a complement to objective clinical measures, though this is rarely done in most European centers. 11

In an effort to address psychosocial issues, several hemophilia-specific questionnaires have been developed (e.g., QoL indexes and activity scores), with numerous investigations focusing on the assessment of patient-reported outcomes (PROs) of PWH worldwide. 12 13 With the advances in available treatments and improved illness control for younger generations, there is an increased interest in the assessment of PROs. As a matter of fact, these are more sensitive and reliable indicators of the impact of hemophilia and the effectiveness of treatment, thus allowing to complement objective clinical assessment with the patient's perspective. 5 However, despite the amount of investigations centered on these issues, no such data have been comprehensively assessed and reported in Portugal, wherein there is an estimated prevalence of 700 cases of hemophilia. 14

The purpose of this investigation is to provide a comprehensive joint description of sociodemographic, clinical, and psychosocial characteristics of PWH in Portugal, to fill an important gap on such knowledge. This is particularly relevant in chronic conditions such as hemophilia that are associated with significant disability, demanding lifelong treatments and high health care costs. Therefore, assessing patients' perspectives on illness symptoms, psychosocial impact and treatment effectiveness is crucial to inform physicians and policymakers toward the improvement of comprehensive health care policies. 7

## Material and Methods

### Design

This is a cross-sectional observational study among PWH registered in the Portuguese Haemophilia Association (APH). An envelope containing an invitation to participate and the description of study objectives, in addition to the informed consent and the questionnaires, was sent by mail to a total of 500 male persons registered in APH as having hemophilia. The surveys were sent in October 2016 and received until May 2017. A telephone call was made to all PWH who had not sent the survey after 3 months (January 2017), both as a reminder and as a mean to clarify possible doubts. Approval for this study was obtained by the Ethical Committee at University of Minho and by the Portuguese Data Protection Agency, and it is registered at clinicaltrials.gov (NCT02870114). Informed consent was obtained from the participants or legal guardians.

### Participants and Procedure

Participants included in this survey were male PWH with hemophilia A or B of all ages. Exclusion criteria were inability to read and write or to consent voluntary participation.

To comprehensively assess PWH of all ages, four similar but age-appropriate versions were developed, varying according to target age (adults vs. teenagers and children) and mode of response (self-report vs. proxy): (1) adults (self-report)— ≥ 18 years old; (2) children/teenagers (self-report)—from 10 to 17 years old; (3) parents (proxy version)—from 6 to 9 years old; and (4) parents (proxy version)—from 0 to 5 years old. Survey content was based on an extensive literature review focused on studies and surveys conducted with PWH worldwide, being further discussed in meetings with medical experts and later refined to guarantee relevance and avoid repetition and respondents exhaustion. The resulting versions were submitted to pilot testing with six adults and six children/teenagers with hemophilia, and four parents/caregivers for comments on content, wording, and length. This process resulted in additional modifications until a final form was reached for each survey version. The variables and questionnaires included in each version are summarized in Table 1 and a more detailed description of the instruments can be found in the following section. Questionnaires that were not yet adapted to European Portuguese (A36 Hemofilia-QoL, CHO-KLAT, HAL, and PedHAL) underwent a complete translation–back translation process followed by pilot testing.

**Table 1:** Survey versions, variables, and questionnaires

Variables	Measures	Adults Age ≥ 18 y	Children/ Teenagers Age 10–17 y	Children Age 6–9 y	Children Age 1–5 y
		Self-Report		Proxy	
Sociodemographic	Sociodemographic questionnaire	✓	✓	✓	✓
Clinical	Clinical questionnaire	✓	✓	✓	✓
Physical activity and sports	Physical activity questionnaire	✓	✓	✓	✓
Pain	Pain questionnaire	✓	✓	✓	✓
Quality of life	A36 Hemofilia-QoL	✓	–	–	–
	CHO-KLAT	–	✓	✓	–
Functionality	HAL	✓	–	–	–
	PedHAL	–	✓	✓	–
Emotional distress	PROMIS Anxiety	✓	–	–	–
	PROMIS Depression	✓	–	–	–
Illness perceptions	IPQ-R	✓	✓	–	–

Abbreviations: CHO-KLAT, Canadian Haemophilia Outcomes-Kids' Life Assessment Tool; HAL, Haemophilia Activities List; IPQ-R, Illness Perception Questionnaire-Revised; PedHAL, Pediatric Haemophilia Activities List; PROMIS, Patient-Reported Outcomes Measurement Information System.

### Data Collection

- Sociodemographic and Clinical Questionnaires: gather information on sociodemographic (e.g., age, education, household, professional status) and clinical (e.g., disease type and severity, inhibitor status, factor replacement consumption, bleeding episodes) characteristics.
- Physical Activity Questionnaire: collects information about physical activity and sports participation.
- Pain Questionnaire: assesses hemophilia-related pain through a set of questions regarding duration, frequency, location, and impact.
- A36 Hemofilia-QoL: 15 evaluates hemophilia-specific QoL through 36 items, according to 9 subscales: physical health, daily activities, joints, pain, treatment satisfaction, treatment difficulties, relationships and social activity, emotional functioning, and mental health. Global QoL raw score ranges between 0 and 144, with higher values indicating better QoL. A percentile score can also be computed (0–100 percentile) for the global scale and each of the dimensions.
- Canadian Haemophilia Outcomes-Kids' Life Assessment Tool (CHO-KLAT): 16 measures hemophilia-specific QoL in children and teenagers through 35 items, which can be answered by the child/teenager with hemophilia or by a proxy (parent version). The total score is presented on a 0 to 100 scale, with a value of 100 being the best possible QoL.
- Haemophilia Activities List (HAL): 17 evaluates patients' self-perceived functional ability, namely, the difficulty in performing activities due to hemophilia. It has 42 items and is divided in 7 subscales: lying/siting/kneeling/standing, function of the legs, function of the arms, use of transportation, self-care, household tasks, and leisure activities and sports. A normalized score can be computed for each dimension and for the global scale, ranging from 0 (worst functional status) to 100 (best functional status).
- Pediatric Haemophilia Activities List (PedHAL): 18 this is an adaptation of the above-mentioned HAL for use with children and teenagers, assessing the same dimensions with 53 items, but with some adjustments to younger ages. A proxy version is also available, to be answered by parents or caregivers. Similarly with HAL, the raw score for each domain is converted to a normalized score, with 0 representing the worst and 100 the best possible functional status.
- PROMIS-Anxiety and Depression (version 1.0): 19 each measure has four items that assess symptoms of anxiety and depression such as fear (anxiety) or hopelessness (depression). Scores range from 4 to 20, with higher scores indicating more severe symptoms. A score of 8 has been proposed as the cut-off for clinically relevant symptoms in both scales. 20
- Illness Perception Questionnaire-Revised (IPQ-R): 21 evaluates illness perceptions and beliefs about hemophilia through 21 items, divided in 7 dimensions: timeline acute/chronic, timeline cyclical, consequences, personal control, treatment control, illness coherence, and emotional representation. Scores vary between 3 and 15 and higher values translate more threatening illness perceptions.

### Data Analysis

Data analysis was conducted using the IBM SPSS version 24 software (Chicago, Illinois, United States). Data were analyzed using frequencies and are described as *n* (%). Continuous variables are presented as mean (M) and standard deviation (SD) and/or median (Md) and range.

## Results

### Return Rate

**Fig. 1:**
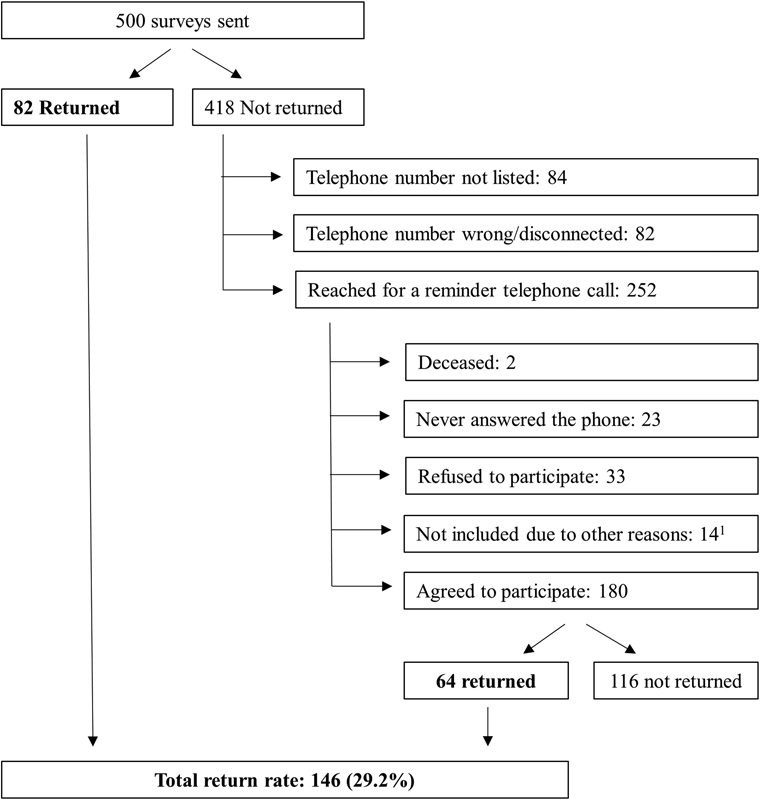
Flowchart of study participants and reasons for nonparticipation. ^1^ Other reasons for nonparticipation included living abroad ( *n* = 6), unable to answer due to other comorbidities (blindness, cerebral palsy, and cognitive impairment) ( *n* = 3), bad phone reception despite several calls ( *n* = 3), and increased factor activity level (>40%) due to liver transplant ( *n* = 2).

Of the 500 surveys sent, 146 were returned by participants (29.2% response rate). Although participants were reminded through telephone call, it was not possible to reach to all PWH. Fig. 1 shows a flowchart where reasons associated with nonparticipation are described. Respondents were not significantly different from nonrespondents in terms of age.

### Demographic Characteristics

Among the 146 participants, 106 (72.6%) were adults (mean age = 43.49; SD = 13.89), 21 (14.4%) children/teenagers between 10 and 17 years old (mean age = 14.00; SD = 2.39), 11 (7.5%) children between 6 and 9 years old (mean age = 7.73; SD = 1.01), and 8 (5.5%) children between 1 and 5 years old (mean age = 3.38; SD = 1.60). All participants were of Portuguese nationality. Concerning geographic distribution, 62 (42.5%) participants were from the north of the country, 38 (26%) from the center, 43 (49.2%) from the south, and 10 (6.9%) from the autonomous regions (results not shown).

All respondents in the proxy version were parents (mean age: 39.47; SD = 5.99), the majority being the mother (17; 89.5%). Most parents were married (15; 78.0%), 3 (15.8%) were single, and 1 (5.3%) was divorced (results not shown). Further sociodemographic characteristics of study respondents are summarized in Table 2 .

**Table 2:** Sociodemographic information of survey respondents

	Adults ≥ 18 y *N* = 106	Children/Teenagers 10–17 y *N* = 21	Children (proxy version)	
			6–9 y *N* = 11	1–5 y *N* = 8
Age	43.49 (13.89) Md = 44 range: 18–74	14.00 (2.39) Md = 14 range: 10–17	7.73 (1.01) Md = 8 range: 6–9	3.38 (1.60) Md = 3.50 range: 1–5
Education (completed level)				
Primary school (1st–4th grade)	6 (5.7%)	12 (57.1%)	–	–
Middle school (5th–9th grade)	25 (23.6%)	9 (42.9%)	–	–
High school (10th–12th grade)	40 (37.7%)	–	–	–
College degree	29 (27.3%)	–	–	–
Postgraduate degree	5 (4.7%)	–	–	–
Marital status				
Single	37 (34.9%)	–	–	–
Married	61 (57.5%)	–	–	–
Divorced/separated	4 (3.8%)	–	–	–
Widower	1 (0.9%)	–	–	–
Professional status				
Student	9 (8.4%)	21 (100%)	10 (90.9%)	–
Full or part-time job	57 (53.7%)	–	–	–
Unemployed	8 (7.5%)	–	–	–
Retired	28 (26.4%)	–	–	–
Medical leave	2 (1.9%)	–	–	–
If unemployed/retired/medical leave a	*N* = 38			
Due to hemophilia	20 (52.6%)	–	–	–
Perceived impact of hemophilia on work b	*N* = 57			
No impact	28 (49.1%)	–	–	–
Little impact	17 (29.8%)	–	–	–
Some impact	9 (15.8%)	–	–	–
High impact	3 (5.3%)	–	–	–
Very high impact	0	–	–	–
Work/school/kindergarten absences due to hemophilia c	*N* = 66 28 (42.4%)	*N* = 21 12 (57.1%)	*N* = 10 8 (80.0%)	*N* = 7 6 (85.7%)
Number of days missed last year	28.57 (55.67) Md = 15 range: 1–293	5.58 (4.46) Md = 3.5 range: 1–15	8.50 (9.20) Md = 4.5 range: 2–30	8.83 (10.76) Md = 4 range: 2–30
Household income				
≤ 530€	11 (10.4%)	1 (4.8%)	1 (9.1%)	0
531€–1,060€	25 (23.6%)	4 (19.0%)	1 (9.1%)	1 (12.5%)
1,061€–2,120€	41 (38.8%)	10 (47.6%)	5 (45.5%)	6 (75.0%)
≥ 2,121€	13 (12.3%)	2 (9.6%)	2 (18.2%)	0
Decline to answer	14 (13.2%)	3 (14.3%)	2 (18.2%)	1 (12.5%)
Perceived impact of hemophilia on finances				
No impact	44 (41.5%)	–	–	–
Little impact	26 (24.5%)	–	–	–
Some impact	16 (15.1%)	–	–	–
High impact	8 (7.5%)	–	–	–
Very high impact	8 (7.5%)	–	–	–

Note: Continuous variables are presented as mean (SD), median (Md), and range. Categorical variables are presented as *n* (%).

a Sample of participants reporting being unemployed, retired, or on medical leave.

b Only assessed among participants who reported a full- or part-time job.

c Number of participants reporting absences due to hemophilia. Only assessed among participants who were working/studying or going to kindergarten.

### Impact of Hemophilia on Employment, Education, and Economic Issues

Table 2 shows that 57 (53.7%) adults had a professional activity and 38 (35.8%) were unemployed, retired, or on medical leave. About half (20; 52.6%) of the participants in this latter situation stated it was due to hemophilia. Among the adult participants who had an occupation, either a full or a part-time job, or a student status, 28 (42.4%) reported missed days from work or school due to hemophilia (M = 28.57; SD = 55.67) and of those on a full or a part-time job, 29 (50.9%) indicated an impact of hemophilia on their professional activity. Most children and teenagers from the 10 to 17 (12; 57.1%), 6 to 9 (8; 72.7%), and 1 to 5 (6; 85.7%) age groups also reported missed days from school. Moreover, 58 (54.6%) adults pointed to an impact of hemophilia on their economic situation (finances).

Most parents answering the proxy versions were employed (13; 68.5%) and 4 (21.1%) were unemployed. From the latter, three (75%) considered that they were in that situation due to their child's hemophilia. Fifteen parents (79%) reported that the time dedicated to their professional activity is limited by hemophilia (no impact: 3 [15.8%]; little impact: 9 [47.4%]; high impact: 4 [21.1%]; very high impact: 2 [10.5%]), and 9 (60%) had missed days from work (M = 9.89; SD = 11.68) to assist the child in issues related to hemophilia (results not shown).

### Clinical Characteristics

Table 3 reveals that most participants in all four age groups had hemophilia A, with severe hemophilia being the most commonly reported (see Table 3 for comparison between age groups). Prophylaxis treatment was reported by 35 (33%) adults, 16 (76.2%) children/teenagers, 9 (81.8%) children between 6 and 9 years, and 4 (50%) children between 1 and 5 years. The occurrence of bleeding episodes in the previous year was reported by 71 (67%) adults, 15 (71.4%) children /teenagers, and 6 (54.5%) and 4 (50%) young children, with the mean number of bleeding episodes varying from 4.53 (SD = 3.36) in the group of children/teenagers to 14.94 (SD = 16.90) among adults.

**Table 3:** Clinical characteristics of survey respondents

	Adults ≥ 18 y *N* = 106	Children/Teenagers 10–17 y *N* = 21	Children (proxy version)	
			6–9 y *N* = 11	1–5 y *N* = 8
Type of hemophilia				
Hemophilia A	89 (84.0%)	19 (90.5%)	10 (90.9%)	8 (100%)
Hemophilia B	17 (16.0%)	2 (9.5%)	1 (9.1%)	0
Hemophilia severity				
Mild	13 (12.3%)	4 (19.0%)	0	1 (12.5%)
Moderate	33 (31.1%)	2 (9.5%)	3 (27.3%)	2 (25.0%)
Severe	60 (56.6%)	15 (71.4%)	8 (72.7%)	5 (62.5%)
Inhibitors				
Yes	14 (13.2%)	1 (4.8%)	3 (27.3%)	2 (25.0%)
Does not know	27 (25.5%)	1 (4.8%)	1 (9.1%)	2 (25.0%)
Age at the time of diagnosis				
During pregnancy/at birth	12 (11.3%)	3 (14.3%)	4 (36.4%)	5 (62.5%)
First year of life	40 (37.7%)	11 (52.4%)	5 (45.5%)	3 (37.5%)
1–5 y	21 (19.8%)	7 (33.3%)	2 (18.2%)	0
6–10 y	10 (9.4%)	0	0	–
≥ 11 y	13 (12.3%)	0	0	–
Does not know	13 (12.3%)	0	0	0
Prophylaxis treatment	35 (33.0%)	16 (76.2%)	9 (81.8%)	4 (50.0%)
Type of factor concentrate				
Plasma-derived	42 (39.6%)	5 (23.8%)	0	0
Recombinant	33 (31.1%)	11 (52.4%)	10 (90.9%)	6 (75.0%)
Bypassing agent	6 (5.7%)	1 (4.8%)	1 (9.1%)	1 (12.5%)
Does not know	14 (13.2%)	2 (9.5%)	0	0
Urgent hospital visits due to hemophilia a : yes	51 (48.1%)	13 (61.9%)	9 (81.8%)	4 (50.0%)
Number of visits	4.73 (4.44) Md = 3 range: 1–16	3.31 (2.14) Md = 2 range: 1–7	3.89 (3.26) Md = 2 range: 1–10	5.25 (3.76) Md = 5 range: 1–10
Hospitalization due to hemophilia a : yes	12 (11.3%)	4 (19.0%)	2 (18.2%)	2 (25.0%)
Number of days	12.58 (11.73) Md = 7.5 range: 4–39	5.67 (3.22) Md = 7 range: 2–8	1.5 (0.71) Md = 1.5 range: 1–2	5.0 (2.83) Md = 5 range: 3–7
Bleeding episodes a : yes	71 (67.0%)	15 (71.4%)	6 (54.5%)	4 (50.0%)
Number of bleeding episodes	14.94 (16.90) Md = 9 range: 1–84	4.53 (3.36) Md = 3 range: 1–12	5.0 (2.53) Md = 4.5 range: 2–8	6.0 (4.69) Md = 6.5 range: 1–10
Joint deterioration: yes	90 (84.9%)	12 (57.1%)	5 (45.5%)	2 (25.0%)
Number of affected joints	4.16 (2.55) Md = 4 range: 1–10	1.67 (1.16) Md = 1 range: 1–4	1.00 (1.00) Md = 1 range: 1–1	1.50 (0.71) Md = 1.5 range: 1–2
Most affected joint b : yes	72 (67.9%)	8 (38.1%)	3 (27.3%)	0
Knee	37 (51.4%)	1 (4.8%)	0	0
Ankle	29 (40.3%)	3 (14.3%)	2 (18.2%)	0
Elbow	14 (19.4%)	4 (19.0%)	1 (9.1)	0
Hip	6 (8.3%)	0	0	0
Shoulder	3 (4.17%)	0	0	0
Comorbidities				
HIV	14 (13.5%)	–	–	–
Hepatitis C	25 (24.0%)	–	–	–
Pain due to hemophilia				
In the previous year	82 (77.4%)	16 (76.2%)	9 (81.8%)	4 (50.0%)
Lasting over 3 months	65 (61.3%)	13 (61.9%)	6 (54.5%)	2 (25.0%)
More than once a week	43 (40.6%)	2 (9.5%)	1 (9.1%)	0
Pain with more impact	N = 82	N = 16	N = 9	N = 4
Ankle	31 (37.8%)	7 (43.9%)	5 (55.5%)	1 (25.0%)
Knee	30 (36.30%)	2 (12.5%)	3 (33.3%)	0
Elbow	8 (97%)	3 (18.8%)	1 (11.1%)	1 (25.0%)
Shoulder	5 (6.1%)	0	0	0
Hip	5 (6.1%)	0	0	0
Others	3 (3.6%)	4 (25.1%)	0	2 (50.0%)
Pain duration c (months)	137.70 (136.46) Md = 96 range: 1–612	28.67 (38.03) Md = 6 range: 1–108	18.00 (22.03) Md = 8.5 range: 1–60	4.50 (0.71) Md = 4.50 range: 4–5
Pain frequency c				
Always present (a)	12 (14.6%)	0	0	0
Daily (b)	15 (18.3%)	0	0	0
Weekly (c)	12 (14.6%)	1 (6.3%)	0	0
Only during movement (d)	21 (25.6%)	4 (25.0%)	4 (44.4%)	0
Bleeding episodes only (e)	9 (11.0%)	6 (37.5%)	4 (44.4%)	4 (100%)
(c) + (d)	2 (2.4%)	0	0	0
(c) + (d) + (e)	3 (3.7%)	0	0	0
(d) + (e)	8 (9.8%)	5 (31.3%)	1 (11.1%)	0

Notes: Continuous variables are presented as mean (SD), median (Md), and range. Categorical variables are presented as *n* (%).

a Data reporting to the previous year.

b More than one response option is possible.

c Concerning the pain with more impact.

Table 3 also gives information on joint deterioration and number of affected joints, showing that most adults (72; 67.9%) reported at least a joint that was most affected as a result of hemophilia. This specific outcome was less pointed out by younger groups (10–17 years: 8 [38.1%]; 6–9 years: 3 [27.3%]; 1–5 years: 0). Further disease and treatment characteristics are detailed in Table 3 , such as information regarding type of factor concentrate, inhibitors status, urgent hospital visits, and hospitalizations due to hemophilia.

Finally, Table 3 exhibits information about pain due to hemophilia, showing that in the previous year pain was reported by a vast majority of participants of all age groups (≥18 years: 82 [77.4%]; 10–17 years: 16 [76.2%]; 6–9 years: 9 [81.8%]; 1–5 years: 4 [50%]). Pain in the lower limbs was considered to have the greatest impact, namely, in the ankles (≥18 years: 31 [37.8%]; 10–17 years: 7 [43.9%]; 6–9 years: 5 [55.5%]; 1–5 years: 1 [25%]). Pain showed a wide duration range, varying from 1 month in three age groups to 612 months (51 years) in the adults group.

### Physical Activity and Sports

As shown in Table 4 , the practice of regular physical activity was reported by 29 (27.4%) adults, 12 (57.1%) children/teenagers, 9 (81.8%) children in 6 to 9 years group, and 4 (50%) children in 1 to 5 years group. In addition to the performance of a regular activity, participants also indicated an activity or sport which they would like to practice, but could not due to hemophilia. Swimming was the most practiced regular physical activity among all age groups, whereas football was the first aspired sport among all age groups. Further details on regular and aspirational activities and sports are described in Table 4 .

**Table 4:** Physical activity practice and aspirational sports of survey respondents

Adults ≥ 18 y ( *N* = 106)		Children/Teenagers 10–17 y ( *N* = 21)		Children (proxy version)			
				6–9 y ( *N* = 11)		1–5 y ( *N* = 8)	
**Regularly:**	29 (27.4%)	**Regularly:**	12 (57.1%)	**Regularly:**	9 (81.8%)	**Regularly:**	4 (50.0%)
1. Swimming	16	1. Swimming	5	1. Swimming	7	1. Swimming	4
2. Walking	5	2. Football	3	2. Hockey	1	2. Football	1
3. Cycle	3	3. Dance 3. Gym	2 2	3. Dance	1	–	–
**Aspirational sport:**	61 (57.5%)	**Aspirational sport:**	15 (71.4%)	**Aspirational sport:**	9 (81.8%)	**Aspirational sport:**	1 (12.5%)
1. Football	24	1. Football	5	1. Football	8	1. Football	1
2. Cycle	12	2. Martial arts	3	2. Swimming	1	–	–
3. Martial arts	5	3. Basketball 3. Handball	2 2	–	–	–	–

### Quality of Life and Functionality

Regarding QoL, Table 5 shows that A36 Hemofilia-QoL global mean score was 96.45 (SD = 27.33), with subscale scores for each specific domain also being reported. Considering CHO-KLAT, mean scores were 75.63 (SD = 12.06) for the 10 to 17 years old group and 76.32 (SD = 11.89) for the 6 to 9 years old group.

**Table 5:** Psychosocial and functionality variables among survey respondents

Measure (range)	Adults ≥ 18 y *N* = 106		Children/Teenagers 10–17 y *N* = 21		Children (proxy version) 6–9 y *N* = 11	
	M (SD)	Min-max	M (SD)	Min-max	M (SD)	Min-max
A36 Hemofilia-QoL						
Global score (0–144)	96.45 (27.33)	31–144	–	–	–	–
Physical health (0–32)	21.57 (6.70)	7–32	–	–	–	–
Daily activities (0–16)	9.99 (5.13)	0–16	–	–	–	–
Joint damage (0–12)	6.64 (2.99)	0–12	–	–	–	–
Pain (0–8)	4.61 (2.23)	0–8	–	–	–	–
Treatment satisfaction (0–8)	5.98 (1.64)	1–8	–	–	–	–
Treatment difficulties (0–16)	11.74 (3.67)	0–16	–	–	–	–
Emotional functioning (0–20)	12.11 (5.21)	0–20	–	–	–	–
Mental health (0–12)	7.85 (2.97)	0–12	–	–	–	–
Relationships and social activity (0–20)	15.18 (4.85)	0–20	–	–	–	–
CHO-KLAT (0–100)	–	–	75.63 (12.06)	54–93	76.32 (11.89)	52–87
HAL/PedHAL (0–100)						
Global score	66.29 (25.47)	5–100	92.35 (11.98)	54–100	86.28 (18.66)	37–100
Lying/sitting/kneeling/standing	60.53 (28.72)	3–100	90.76 (15.16)	46–100	86.63 (19.73)	34–100
Functions of the legs	55.92 (30.21)	0–100	89.30 (19.39)	20–100	78.80 (20.26)	44–100
Functions of the arms	69.63 (27.51)	0–100	94.60 (8.53)	77–100	91.52 (20.99)	30–100
Use of transportation	68.94 (29.55)	13–100	94.13 (18.37)	20–100	88.48 (20.02)	33–100
Self-care	80.69 (22.89)	8–100	96.51 (8.51)	67–100	88.91 (21.79)	28–100
Household tasks	74.76 (25.45)	0–100	96.83 (7.19)	80–100	90.30 (20.52)	33–100
Leisure activities and sports	70.49 (25.52)	0–100	90.04 (13.61)	60–100	85.71 (21.32)	42–100
PROMIS (4–20)						
Anxiety	7.02 (2.95)	4–19	–	–	–	–
Depression	6.23 (3.26)	4–20	–	–	–	–
IPQ-R (3–15)						
Timeline acute/chronic	12.95 (2.06)	7–15	12.25 (2.73)	6–15	–	–
Timeline cyclical	9.46 (2.65)	3–15	9.85 (3.17)	3–15	–	–
Consequences	8.72 (3.12)	3–15	7.70 (2.54)	4–14	–	–
Personal control	7.71 (2.46)	3–15	8.15 (2.11)	4–14	–	–
Treatment control	6.79 (2.09)	3–15	5.10 (1.55)	3–7	–	–
Illness coherence	5.69 (2.42)	3–15	6.25 (2.45)	3–11	–	–
Emotional representation	6.62 (3.22)	3–15	7.50 (4.19)	3–15	–	–

Abbreviations: CHO-KLAT, Canadian Haemophilia Outcomes-Kids' Life Assessment Tool; HAL, Haemophilia Activities List; IPQ-R, Illness Perception Questionnaire-Revised; PedHAL, Pediatric Haemophilia Activities List; PROMIS, Patient-Reported Outcomes Measurement Information System.

Results from HAL and PedHAL are also presented in Table 5 revealing that, in what concerns functionality, PWH of all ages had the lowest scores on “Functions of the legs.” Adults reported better functioning in “Self-care” (M = 80.69; SD = 22.89), while children/teenagers had the highest scores on “Household tasks” (M = 96.83; SD = 7.19) and children in 6 to 9 years old group reported less difficulties in “Functions of the arms” (M = 91.52; SD = 20.99).

### Emotional Distress

Mean scores of psychological distress were 7.02 (SD = 2.95) for anxiety and 6.23 (SD = 3.26) for depression ( Table 5 ). Considering the proposed cut-off scores of 8 for clinically relevant symptoms, 37 (36.7%) adults had clinically relevant anxiety symptoms and 28 (27.2%) had significant depression symptoms (results not shown).

### Illness Perceptions

The highest scores on IPQ-R, presented in Table 5 , were found on the “Timeline acute/chronic” dimension, both for adults (M = 12.95; SD = 2.06) and children/teenagers (M = 12.25; SD = 2.73), followed by the “Timeline cyclical” dimension, which also scored high for both groups (M = 9.46; SD = 2.65 for adults and M = 9.85; SD = 3.17 for children/teenagers).

## Discussion

This was the first nationwide hemophilia study conducted in Portugal simultaneously covering sociodemographic and clinical characteristics, as well as functional and psychosocial variables.

Findings revealed a high prevalence of joint deterioration and pain, with the ankles and the knees being the most affected joints among all age groups. A significant impact of hemophilia on professional and economic levels was particularly evident. Moreover, significant anxiety and depression symptoms were found on 36.7% and 27.2% of adults, respectively, and a belief of chronicity and symptoms unpredictability was particularly prominent among adults and children/teenagers. QoL was moderately affected among adults but less affected in teenagers and children.

### Socioeconomic Impact: Employment, Education, and Economic Issues

In this study, 38 participants were currently not working (unemployed, retired, or on medical leave), with approximately half of them claiming to be in that situation due to hemophilia. Nevertheless, and concerning particularly unemployment figures (7.5%), these were similar to current global rates in Portugal (7.7%), showing that PWH in this sample do not experience higher unemployment than the general male population. 22 Moreover, in this sample, among the adults who were working, 50.9% declared an impact of the disease on their professional activity. Regardless of the professional status, 54.6% of adults further pointed to an impact of hemophilia on their financial income, underlining the relevance of further exploring the individual and household economic burden of the disease, which has been an overlooked subject in this field. Parents of children with hemophilia also reported missed days from work and an impact of their child's disease on their work life, similarly with surveys conducted in other countries. 23 24 School absenteeism was also a relevant issue for teenagers and children, since more than half of participants reported missed days from school due to hemophilia. Despite the lack of evidence of a negative impact of hemophilia on educational status of PWH, 25 Shapiro et al 26 observed that children with higher bleeding rates missed more days of school and tended to have lower academic achievement. Among adults, work/school absences were reported by 42.4% participants and could last up to 293 days. These findings unequivocally illustrate the negative impact of hemophilia on work- or school-related activities, corroborating the data from other surveys. 13 23 24 However, it has already been shown that prophylactic treatment can effectively reduce the number of school and work absences, 27 28 suggesting that there might be some room for improvement in Portugal, particularly among adults, who were less often submitted to prophylactic regimens.

Since employment status and perceived impact of hemophilia have been shown to negatively influence clinical and psychosocial outcomes, 29 a special attention should be given to these socioeconomic issues, by considering more flexible educational and employment policies that could benefit PWH. Interestingly, the number of participants with college education (27.3%) in this current sample is similar to the Portuguese general population (23.9% in the 25–64 age group), 30 showing that the impact of hemophilia is not reflected on lower educational achievement. More robust conclusions concerning this issue should be sought in future studies, by comparing patients of different hemophilia severities.

### Clinical Characteristics: Bleeds, Prophylaxis, and Pain

To provide a comprehensive overview of hemophilia, people of all disease severities were included in this survey. Distribution by type of hemophilia was in line with general prevalence estimations, stating that hemophilia A affects 80 to 85% of the hemophilia population. 5

Due to the occurrence of repeated joint bleeds, joint deterioration is the hallmark of hemophilia, posing a relevant burden even in mild patients. 31 According to other studies, 24 a higher number of bleeding episodes were reported by adults than by teenagers and children, illustrating the positive impact of early prophylaxis versus on-demand therapy in decreasing the number of bleeds. 4 This discrepancy among age groups also occurred regarding the report of affected joints. While 67.9% of adults reported at least a joint that was most affected as a result of hemophilia, this outcome was less pointed out by the 10 to 17 (38.1%) and the 6 to 9 (27.3%) years old groups. Nonetheless, the number of reported bleeds and presence of affected joints in the younger age groups is still very significant, considering that most participants are under prophylaxis. Though these data could be partially explained by some over reporting from parents in the proxy versions, future studies should seek to clarify this issue to effectively prevent the development of debilitating hemophilic arthropathy in these age groups.

In the current study, 33% of adults reported to be under prophylaxis treatment. These figures are notably higher than results regarding countries such as Algeria (0%), China (4%), and Argentina (14%), though below other countries such as France (58%), Italy (56%), Germany (48%), and Canada (48%), according to the HERO study, 32 an international survey conducted among PWH in several countries. However, regarding these figures, hemophilia severity was not taken into account and thus caution must be applied when establishing comparisons. For instance, if only the severe cases of current adult sample were considered, prophylaxis would rise to 55%. Nevertheless, and according to a recent study on prophylaxis access among severe PWH, 33 these Portuguese figures are still below other countries, such as Belgium, Ireland, and the Netherlands, wherein 76 to 100% of adults have access to prophylaxis. Concerning Portuguese children and teenagers, access to prophylaxis is higher when compared with adults, covering 56 to 100% of them, which matches the practices of developed countries like Belgium, Finland, Ireland, or Norway, for instance. 33

Concerning pain, current findings confirm that this is a critical aspect of hemophilia, by revealing a high prevalence of pain at all age levels. Though comparisons of pain reports across studies are constrained by the variety of methods used, this is in accordance with the high prevalence of pain among PWH reported by other studies. 24 34 Present findings further highlighted that pain constitutes part of the daily life of a majority of Portuguese PWH, since adults revealed a high duration and frequency of pain, which could last for more than 50 years and occur on a daily or weekly basis or even constantly. Pain experience in hemophilia is associated with worst psychological functioning, functional disability, and diminished QoL, additionally increasing the burden of the disease itself. 34 35 The development of evidence-based pain management guidelines and effective practices is therefore imperative and must be a priority in hemophilia care. In this scope, three issues are crucial and should be considered: a closer collaboration between hemophilia clinicians and pain specialists; the implementation of effective pain control strategies that could include, beyond pharmacological, nonpharmacological approaches, namely, psychological strategies which cost-effectiveness has been shown across distinct diseases 36 despite its underuse among PWH; and, finally, the recognition of pain as a critical hemophilia issue by hemophilia health care providers.

### Physical Activity and Sports

Physical activity has many benefits and is recommended for PWH as a means of promoting physical fitness, healthy neuromuscular development, and social and psychological well-being, 5 being even associated with a decrease in pain perception and in factor replacement consumption. 37

A high frequency of physical activity among PWH has been reported elsewhere; 38 however, only 27.4% of adults in this Portuguese sample declared practicing sports regularly. In fact, these results match the Portuguese global pattern, with recent reports indicating that 64% of the general population never exercise or play sports. 39 Nonetheless, the hypothesis can be made that, in some cases, low physical activity frequency is explained by fear of bleeding episodes, underlining the need to promote knowledge about the role of physical activity among PWH, as well as specific recommendations and protective measures. Actually, in a recent study, 40 the main reasons indicated by PWH for not doing any sport were their physical condition and fear of hurting themselves.

Among younger PWH, about half in the 10 to 17 (57.1%) and 1 to 5 (50%) years old group, and most in the 6 to 9 years old group (81.8%), declared to practice physical activity regularly. This corroborates other findings showing a high proportion of children with hemophilia practicing regular sports, 38 with activity levels comparable to healthy peers. 41 Similar to other studies with PWH, the most desired activity was football, in all age ranges, 24 and the most commonly practiced sport was swimming, which is generally recommended to PWH as a low-impact sport. 42 Concerning this issue, the most important is to implement strategies focused on fostering regular engagement in physical activity among PWH, promoting education about its benefits and potential risks, as well as timely and adequate measures to deal with eventual injuries. 5 40 42

### Quality of Life and Functionality

Studies generally report diminished QoL among PWH 9 10 43 even in mild patients, 44 though some studies account for QoL scores in PWH comparable to the general population when considering specific age groups 45 or only mild/moderate patients. 46 In the current study, QoL scores of Portuguese adults fall in the 50 to 75 percentile, translating a moderate level of health-related QoL in all dimensions considered. In fact, these results are in accordance with QoL scores described in a culturally similar sample from Spain, using the same questionnaire. 15 Interestingly, an investigation in Iran, 47 also using this questionnaire, revealed lower mean scores, with some dimension scores falling below the 50% percentile and the global QoL percentile being 49%. A possible explanation for this might be that Iran is a developing country, likely exhibiting a limited access to prophylaxis, 48 along with more social and economic constraints which potentially moderate negatively the impact of the disease on QoL. In addition, these comparisons also support the positive impact of prophylaxis on improving QoL 6

Among children and adolescents with hemophilia, QoL is generally less affected, 49 50 albeit decreased QoL scores have also been reported when compared with healthy peers. 51 In the present study, CHO-KLAT mean scores are slightly lower than those reported in the Netherlands 49 and Canada, 50 higher than those found in Brazil 52 and Germany, 49 but broadly consistent with those obtained in France, United Kingdom, and Spain. 49

Furthermore, regarding functionality assessment, it was concluded that “Functions of the legs” was the lowest scoring dimension in all age ranges, congruently with the ankles and the knees being reported as most affected joints and painful locations, and matching other studies regarding functionality. 18 34

### Emotional Distress and Illness Perceptions

Concerning emotional distress, 36.7 and 27.2% of adults revealed clinically relevant anxiety and depression symptoms, respectively, in accordance with other studies among PWH describing rates of 22 to 37% of participants with significant distress. 24 29 53 Regarding these figures, it is important to highlight the well-known influence of psychological factors on both pain experience and QoL in PWH. 35 Indeed, it was previously noted that changes in QoL are better explained by psychosocial, rather than clinical predictors, 25 highlighting psychosocial health as a priority target in the improvement of health status and QoL in PWH. 12 24 An important implication stemming from this is that comprehensive care teams should be multidisciplinary and include a complete assessment of psychosocial status and psychological support, contributing to an integrated disease management plan. Furthermore, since psychological factors are amenable to change or to active management through psychological interventions, there is a need to advance knowledge in this field, through the implementation of interventions targeting psychological aspects of PWH, which effectiveness should be evaluated.

Besides emotional characterization, the evaluation of illness beliefs was an innovative feature of this survey. Both adults and children/teenagers had the highest scores on “Timeline acute/chronic” dimension, indicating that participants had the perception of this being a lifelong disease, deemed as chronic and permanent rather than acute or temporary. Moreover, the second highest score on “Timeline cyclical” dimension shows how participants consider hemophilia symptomatology as unpredictable, unstable, and hard to manage. Given the impact of illness beliefs on treatment adherence by PWH, 54 this result underlines the relevance of developing and implementing educative programs that promote knowledge about hemophilia and its symptoms, along with the induction of positive but realistic expectancies on the effect of treatments. Since hemophilia is actually a chronic lifelong disease, yielding unpredictable symptoms, psychosocial interventions in this field should promote acceptance as an adaptive coping disease mechanism and a more positive view of the expectations concerning hemophilia.

### Limitations

This study has some limitations that should be addressed. The representativeness of the participants was not controlled for and the exclusive inclusion of PWH registered on a patients' organization may represent a bias on study sampling. Simultaneously, the low response rate may be responsible for some nonresponse bias that was not controlled for, as there was no information available concerning the characteristics of nonparticipants, except for age. Furthermore, it is also likely that people who participated present distinct psychosocial characteristics, related to disease acceptance, coping strategies, or emotional responses (e.g., sadness, anger), when compared with nonparticipants. Hence, the likelihood of not having captured the whole Portuguese hemophilia picture should be taken into account.

Moreover, some of the data could not be directly compared with other investigations due to relevant variations in clinical features of patients included and the fact that the distribution of variables of interest in those studies is sometimes only described in terms of type of hemophilia, factor activity level, treatment regimen, age, or other characteristics, which confound comparative analysis. Given the global aim of providing a comprehensive description of PWH, the current results were not presented by severity of hemophilia, which may underscore some characteristics of more severe patients, which were a majority in this sample.

## Conclusion

This survey provided the most complete characterization of Portuguese PWH to date, uncovering several intervention needs that are critical to improve well-being and QoL. This allows for a broader and deeper understanding of life with hemophilia and thus contributes to evidence that can be used in providing more effective patient care among PWH. Particularly, this survey represents the first assessment of psychosocial variables among PWH in Portugal, yielding reliable data to inform clinical practice that is not derived from conclusions from other countries. Globally, the results reported on clinical and psychosocial variables are in accordance with studies from other countries and with what might be expected in a developed country with a strong national health care service. Current findings contributed to further underline the relevance of issues that have been receiving increased attention in the hemophilia field, particularly the need to improve pain assessment and management and expand psychosocial care. In fact, according to a recent survey of European countries, pain management and social/psychological support are still among the least available specialty services in hemophilia care and, in Portugal, there was even a reported decrease in access to social and psychological support in recent years. 29

Further detailed analysis of the data are warranted and should consider the results by hemophilia type, severity, and presence of inhibitors, to achieve a deeper understanding of the hemophilia experience of Portuguese patients and better contribute for the improvement of integrated care. Longitudinal studies will also be important to help determine the predictors of disease course and adjustment to tailor interventions that promote QoL and well-being.
